# Employee burnout and positive dimensions of well-being: A latent workplace spirituality profile analysis

**DOI:** 10.1371/journal.pone.0242267

**Published:** 2020-11-17

**Authors:** Laura Dal Corso, Alessandro De Carlo, Francesca Carluccio, Daiana Colledani, Alessandra Falco

**Affiliations:** 1 Department of Philosophy, Sociology, Education and Applied Psychology, University of Padua, Padua, Italy; 2 Giustino Fortunato University, Benevento, Italy; 3 Department of Human Science (Communication, Training, Psychology), LUMSA University, Rome, Italy; Medical University Innsbruck, AUSTRIA

## Abstract

In recent years, a new and promising construct has attracted the attention of organizational research: Workplace spirituality. To investigate the role of workplace spirituality in organizational contexts, two studies were carried out. Study 1 explored the mediation role of workplace spirituality in the relationship between positive supervisor behaviors and employee burnout. Results showed that workplace spirituality strongly contributes to reduce burnout and mediates the effect of supervisor integrity in reducing this threat. Study 2 considered the relationships of workplace spirituality with positive affectivity, resilience, self-efficacy, and work engagement. In particular, workplace spirituality profiles were investigated through latent profile analysis (LPA). Findings showed that workplace spirituality is related to higher positive affectivity, resilience, self-efficacy, and work engagement. In contrast, a workplace spirituality profile characterized by a low-intensity spiritual experience is associated with higher negative feelings. The practical implications of these findings are discussed.

## Introduction

In recent years, the organizational research field has become increasingly interested in workplace spirituality. The emerging literature suggests that workplace spirituality should be considered a multidimensional construct that can be crucial in improving employees’ conditions and organizational performance [1–4]. Despite its allure, there is no consensus on its definition, operationalization, and interpretation. The issue is defining a multi-dimensional and highly subjective construct [5], as well as identifying its possible overlapping with religiousness [6]. Among the many definitions identified in the literature [7], the Kinjerski and Skrypnek’s perspective is particularly suited to organizational contexts. According to it, workplace spirituality—denominated also “spirit at work” by the authors—is a positive state including physical, affective, cognitive, interpersonal, spiritual, and mystical dimensions [3]. Specifically, spirit at work involves a physical state of arousal and energy; feelings of well-being and joy; a cognitive sense of being authentic and engaged in meaningful occupations; interpersonal feelings of connection to others; a positive spiritual connection to something larger than self; and a mystical dimension characterized by a sense of perfection and transcendence [3, 8].

Spiritual workplaces encourage employees’ sense of community, recognize their spiritual-mystical needs, foster feelings of engagement in work, and support integrity, respect, responsibility, and personal growth [3, 9–13].

Overall, research highlighted the positive role of workplace spirituality [14–16] and showed its beneficial effects across different organizational contexts, highly affected by technological innovations [17], that gave rise to new kinds of work structures and innovative working environments and relation sharing [18]. For instance, a positive association was found between workplace spirituality and job satisfaction among employees of the private insurance sector in India [19], whereas in the healthcare setting positive relationships were observed with organizational citizenship behavior, improved quality of resident care, employees’ affective commitment, and reduced absenteeism and turnover intentions [20–23]. Among Australian academic staff members evidence was also provided for a positive link between workplace spirituality and employee well-being, while a negative association was found with occupational stress [24]. Moreover, positive associations were observed between workplace spirituality and organizational performance. For instance, some authors [25] documented a positive relationship between workplace spirituality and performance in employees of the Indonesian banking sector; whereas others [26] observed the same among Indian employees.

Research showed that several variables, concerning both personal and organizational factors, may be considered antecedents of workplace spirituality. With regard to personal factors, a central role was attributed to positive energy, values orientation, and personality traits such as conscientiousness and openness to possibilities [3, 8 27, 28]. In contrast, individual differences, such as age, gender, education, or income, seem to not be related to the experience of spirit at work [27].

Apart from personal characteristics the context plays a crucial role in the development of the spirit at work experience [15, 27, 29]. Research, in particular, demonstrated that organizational factors such as strong organizational foundation, organizational integrity, positive workplace culture, opportunities for personal fulfillment, continuous learning, and regard for employees [28] are crucial antecedents of workplace spirituality. In particular, the literature suggests that supervisor behaviors have a central role [28, 30–32].

Supervisor behaviors may be considered a key organizational factor that can effectively influence all other organizational features leading toward an increase of spirit at work. Research, in particular, showed that supervisors who promote professional and personal growth, maintain organizational integrity, and demonstrate regard for employees’ work, may be considered the main contributors of spirit at work feelings within organizations [27, 33].

Overall, scientific literature converges in attributing a positive role to spirit at work in organizational contexts. However, much research concentrated on Asian contexts, therefore cross-cultural studies are highly recommended to consolidate knowledge on the topic [24]. In addition, further research is needed to better define the features and correlates of workplace spirituality in organizational contexts and its actual role in influencing organizational outcomes [24].

The present paper aims to provide a new contribution to this topic through two studies. The first one explored the role of workplace spirituality between positive supervisor behaviors and employee burnout. The literature showed that supporting workplace spirituality may have a beneficial role in increasing employee well-being [34–37], but only few studies considered its effects on employee burnout [38–42]. In the second study, the relationships of workplace spirituality with a series of other variables relevant in the organizational context were explored. In particular, Latent Profile Analysis (LPA) was performed to identify workplace spirituality profiles based on the four subscales of the Spirit at Work Scale (SAWS) [3, 43]. Finally, the profiles emerged were compared for positive affectivity, resilience, self-efficacy, and work engagement. This study intends to provide a new contribution on the features and correlates of workplace spirituality and aims to reach a deeper knowledge of the construct and its actual role in organizational contexts.

## Study 1

Over the last ten years, the promotion of employees’ physical and psychological well-being has become a central concern for companies and institutions. A promising contribution in this field was provided by positive psychology [44–47], which highlighted the role of positive individual and organizational resources in promoting desirable vocational outcomes [48, 49]. Much research, for instance, showed that positive supervisor behaviors, positive relationships between supervisors and employees, as well as the integrity of organization and supervisor behaviors, are key factors for promoting employee well-being [4, 32, 50–53]. Supervisor behaviors associated with positive outcomes are support, empowerment, trust, confidence, and integrity; conversely, supervisor behaviors such as control or low support were linked with stress [52, 54–58].

As stated previously, research highlighted the positive role of workplace spirituality in the promotion of employee well-being [34–36, 59–61]. However, very few studies considered its effects on burnout [41, 42, 62–65]. Nevertheless, this topic seems relevant because the burnout syndrome is a serious threat to health [66]: it was recently included in the International Classification of Diseases, 11^th^ revision, by the World Health Organization (ICD-11) [67]. Burnout represent a response to chronic emotional and interpersonal stress factors in the workplace; it consists of three dimensions: exhaustion, cynicism and professional inefficiency [68]. Research efforts aimed to identify factors and mechanisms that may attenuate its dangerous effects are always welcome. In addition, exploring the relations between workplace spirituality and employee burnout would be useful to increase awareness of the promising role of spirituality in organizational contexts and to prevent work-related stress and mental health outcomes. In fact, in several countries, burnout syndrome has been recognized as an occupational disease [69].

Taking into account the lack of studies focusing on the role of spirit at work on burnout symptoms, the present study aims to explore the relationships between positive supervisor behaviors and burnout, hypothesizing that workplace spirituality may have a mediating effect in this relationship.

As suggested by the literature, workplace spirituality should be linked to both variables. In particular, research showed that supervisor integrity and responsible behaviors could be viewed as antecedents of workplace spirituality, which, in turn, is an antecedent of employee well-being. Supervisor integrity and responsible behaviors, in addition, were recognized as antecedents of well-being among employees [70]. We, therefore, expect a negative relationship between supervisor integrity and employee burnout, which should be both direct and mediated by spirit at work. Specifically, we hypothesize that the supervisors may reduce employee burnout by showing integrity and by acknowledging their spiritual needs. The study intends to provide new evidence on the role of spirit at work in reducing negative work outcomes while, at the same time, exploring the effects of positive supervisors. Moreover, the work may provide new insights for the development of intervention strategies aimed to improve supervisor stress management competencies.

### Method

#### Participants and procedure

Participants were 315 Italian employees (males = 214; mean age = 43.92 years, SD = 9.84); the majority were administrative employees (67.1%), 15.9% were blue-collars, 11.5% were managers, and 5.4% had other occupations. The education level of the sample was rather high with 84.7% of participants having a high-school or university degree. The majority of participants (92.4%) had a full-time contract (40 hours per week) and a seniority in their company of over ten years (63.9%, up to ten years 36.1%).

Participants were recruited in three different companies (operating in metalworking, banking, large scale retailing). Participants were asked to rate their feelings on workplace spirituality and their burnout levels. They were also asked to rate their supervisor respectful and responsible behaviors, taking into account a series of specific behaviors.

The study was approved by the Ethical Committee for the Psychological Research of the University of Padova. All participants gave written informed consent and were duly informed that participation in was anonymous and voluntary.

#### Measures

The respectful and responsible behavior scale (RR) of the Stress Management Competency Indicator Tool (SMCIT) [71] was used to evaluate positive supervisor behaviors. The scale includes 17 items and assesses three main sets of manager’s competencies: integrity (e.g., “This Manager is a good role model”), managing emotions (e.g., “This Manager’s moods are predictable”), and considerate approach (e.g., “This Manager shows consideration for the team’s work-life balance”). High scores on this scale describe a supervisor characterized by behaviors that are respectful, consistent, and open to communication. The items were scored on a 5-point scale ranging from 1 “strongly disagree” to 5 “strongly agree”.

The 18 items of the Spirit at Work Scale (SAWS) [3, 43] were administered to evaluate workplace spirituality. The instrument assesses the experience of spirituality in the workplace through four main factors: engaging work (e.g., “I am passionate about my work”), sense of community (e.g., “I feel like I am part of ‘a community’ at work”), spiritual connection (e.g., “My spiritual beliefs play an important role in the everyday decisions that I make at work”), and mystical experience (e.g., “I experience moments at work where everything is blissful”). The items were scored on a 6-point scale ranging from 1 “completely untrue” to 6 “completely true”.

The nine-item scale of the *Q*_*u*_*-Bo* test [72] was employed to assess burnout. The scale includes three sub-dimensions, measured by three items each: exhaustion (e.g., “I feel burned out from my work”), cynicism (e.g., “My work has no importance”), and a reduced sense of personal accomplishment (e.g., “I feel incapable of doing my job”). The scale was scored on a 6-point scale ranging from 1 “completely disagree” to 6 “completely agree”.

#### Statistical analyses

Prior to testing the hypothesized structural model, reliability and factor structure of all the instruments were evaluated. Then, structural equation modeling was used to explore the relations between supervisor behaviors, workplace spirituality, and employee burnout. Three latent variables were included in the model. Specifically, respectful and responsible supervisor behaviors were the predictor, workplace spirituality was the mediating variable, and burnout was the outcome. All three latent variables were measured using parcels as indicators (for all scales, parcels were the mean score of subscales) [73] and the maximum likelihood was used as an estimator.

In the mediation model, all paths were estimated and the 95% bootstrap confidence interval (5,000 bootstrapped samples) was used to test the significance of the indirect effect.

To evaluate the model, several goodness-of-fit indices were used: *χ*^*2*^, Comparative Fit Index (CFI) [74], Standardized Root Mean Square Residual (SRMR) [75], and Root Mean Square Error of Approximation (RMSEA) [76]. Concerning *χ*^*2*^, a solution fits the data well when the value is non-significant (*p* ≥ .05). This statistic, however, is sensitive to the sample size. Therefore, inspection of the other fit indices is recommended. In particular, a good fit is supported by CFI indices close to .95 (.90 to .95 for a reasonable fit), SRMR values less than .08, and RMSEA smaller than .06 (.06 to .08 for a reasonable fit) [77–79].

All the analyses were run using the Mplus7.4 package [80].

### Results

Descriptive statistics for all the scales are reported in Table 1. For all instruments, factor structure was confirmed and the reliability was above .60 which is the lower bound for satisfactory reliability in applied research [81–83]. As for the respectful and responsible supervisor behaviors scale of the Stress Management Competency Indicator Tool, a model was tested defining three latent factors (integrity, managing emotions, considerate approach), measured by five to six items each. The model reached an adequate fit (*χ*^*2*^(116) = 302.87, *p* ≅ .00; RMSEA = .08; CFI = .92; SRMR = .05). Regarding the Spirit at Work Scale, the model was tested using items as indicators and four factors were modeled: engaging work, sense of community, spiritual connection, and mystical experience. Fit indices suggested an adequate fit (*χ*^*2*^(129) = 336.88, *p* ≅ .00; RMSEA = .07; CFI = .91; SRMR = .05). The factor structure of the Q_*u*_-Bo burnout scale was also confirmed. The model was tested using items as indicators and three factors were defined: exhaustion, cynicism, and a reduced sense of personal accomplishment. The model reached an excellent fit (*χ*^*2*^(24) = 51.37, *p* ≅ .00; RMSEA = .06; CFI = .96; SRMR = .05). All models were fitted using the maximum likelihood mean adjusted estimator (MLR) [77, 80].

**Table 1:** *N* items, mean, *SD*, alpha coefficients, composite reliability (CR), and average variance extracted (AVE) for all the scales used.

	*N items*	*Mean*	*SD*	*alpha*	*CR*	*AVE*
Integrity	5	3.63	0.85	0.88	0.88	0.60
Managing emotions	6	3.28	0.83	0.87	0.88	0.55
Considerate approach	6	3.38	0.70	0.80	0.82	0.46
**Respectful and responsible supervisor behaviors**	**17**	**3.43**	**0.72**	**0.94**	**0.95**	**0.53**
Engaging work	7	4.05	1.13	0.89	0.89	0.55
Sense of community	3	4.10	1.12	0.75	0.75	0.51
Spiritual connection	3	3.60	1.26	0.75	0.75	0.50
Mystical experience	5	3.77	0.84	0.66	0.67	0.35
**Spirit at work**	**18**	**3.90**	**0.94**	**0.93**	**0.94**	**0.48**
Exhaustion	3	2.33	1.12	0.80	0.83	0.63
Cynicism	3	1.60	1.01	0.87	0.89	0.73
Reduced sense of personal accomplishment	3	1.67	0.95	0.78	0.81	0.60
**Burnout**	**9**	**1.87**	**0.86**	**0.88**	**0.94**	**0.66**

**Fig 1:**
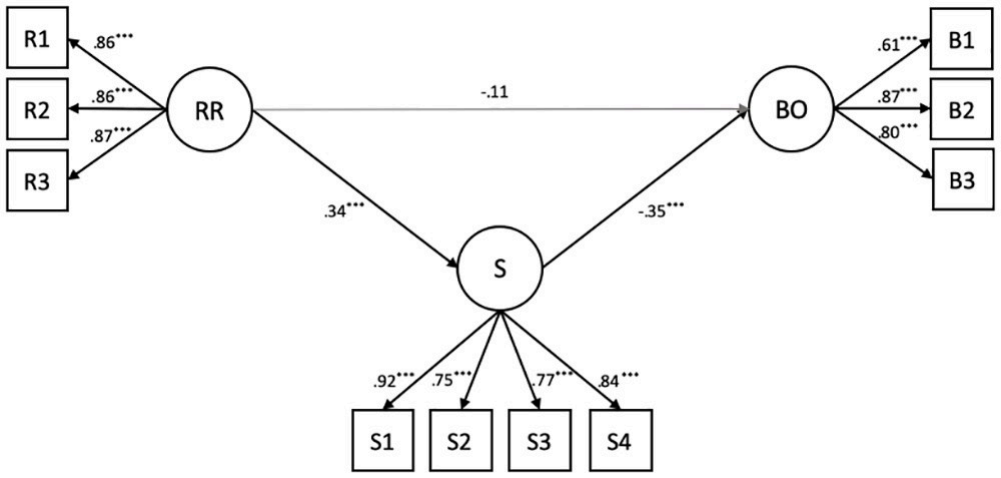
Path diagram for the mediation model. Note. RR = respectful and responsible supervisor behaviors; R1 = integrity, R2 = managing emotions; R3 = considerate approach; S = spirit at work; S1 = mystical experience; S2 = spiritual connection; S3 = engaging work; S4 = sense of community. BO = burnout; B1 = exhaustion; B2 = cynicism; B3 = reduced sense of personal accomplishment. Standardized coefficients; all values are significant *p* ≤ .001 (excluding the effect of RR on burnout 95% *CI*: -0.26, 0.04).

The structural equation model tested to explore the effects of supervisor behaviors and workplace spirituality on employee burnout is represented in Fig 1. The model reached a successful fit: *χ*^*2*^(32) = 92.06, *p* ≅ .000; RMSEA = .08; CFI = .96; SRMR = .06. In the measurement model, all loadings of indicators ranged between .61 and .92, and all correlations between latent constructs were lower than 1 (correlations ranging from .23 and .38, in absolute values), indicating that latent variables represented distinct constructs from both a conceptual and an empirical point of view.

Results of the structural model indicate the negative effects of both spirituality and respectable and responsible supervisor behaviors on employee burnout. However, the effect of supervisor behaviors on employee burnout was only indirect and mediated by spirituality. Specifically, supervisor behaviors had a positive and significant effect on workplace spirituality (95% *CI* = .20, .47), which, in turn, showed a negative effect on burnout (95% *CI* = -.51, -.18). Because supervisor behaviors had a negative, but low and non-significant, direct effect on employee burnout (95% *CI* = -.26, .04), workplace spirituality totally mediated this relationship (95% *CI* = -.20, -.05). In line with expectations, these results showed the relevant role of workplace spirituality in reducing burnout and highlighted the mediating effect of this variable between supervisor behaviors and employee burnout.

### Discussion

This study aimed to explore the mediation role of workplace spirituality in the relationship between positive supervisor behaviors and employee burnout. Results showed that both variables have a positive effect on the reduction of employee burnout. However, the effect of supervisor integrity is only indirect and mediated by spirituality. Results, in other words, suggested that supervisors influence employee burnout through their ability to recognize employees’ spiritual needs [4].

The concept of workplace spirituality has only recently been introduced in organizational research. However, several findings supported its crucial role in influencing a variety of job outcomes across several settings and cultures [19, 34, 84]. Overall, research showed a positive effect of this variable in different vocational outcomes, such as job satisfaction, commitment, job involvement, well-being, productivity [4, 7, 37, 85–87]. Results of the present study, in line with the literature, showed the usefulness of considering employees’ inner needs in the workplace and highlighted the beneficial effect of workplace spirituality in the reduction of negative work outcomes. Workplace spirituality, in fact, had a negative direct effect on burnout and totally mediated the relations between supervisor behaviors and employee burnout. Results, therefore, highlighted the relevance of recognizing employees’ spiritual facets and, at the same time, stressed the crucial role of management competencies in the reduction of burnout symptoms.

## Study 2

As shown in Study 1, workplace spirituality has an effective role in influencing employee well-being. Workplace spirituality may also be associated with other variables affecting individuals’ working life. Exploring these relationships is a meaningful goal because it could provide new insights to develop intervention programs aimed to improve organizational well-being and employee fulfillment. This study aims to explore the relations of workplace spirituality with several variables, which influence the work experience, such as positive affectivity, resilience, self-efficacy, and work engagement. Much research, in fact, underlined the important contribution of these variables in the professional field [88–93]. In particular, the relationships of workplace spirituality with other constructs were explored considering latent workplace spirituality profiles. Specifically, Latent Profile Analysis (LPA) was used to identify groups of individuals showing similar patterns of scores across the four subscales of the Spirit at Work Scale (SAWS) [3, 43]: engaging work, sense of community, spiritual connection, and mystical experience.

LPA is a person-centered analysis, which allows for the classification of participants into groups defined by a similar configuration of scores in a set of variables [94]. In personality psychology, LPA is used to define higher-order typologies that describe individual differences better than individual scale scores [95–98]. In this study, LPA is used to identify groups of individuals characterized by a similar profile in the Spirit at Work Scale (SAWS) [3].

This contribution seems interesting because, to the best of our knowledge, workplace spirituality profiles have never been investigated, even if they may provide useful insights for better knowledge and definition of the construct. Moreover, exploring the relationships of workplace spirituality profiles with other constructs affecting the vocational experience may help to shed light on the actual role of spirituality in the organizational context.

### Method

#### Participants and procedure

Participants were 232 Italian employees (males = 148; mean age = 38.47 years, SD = 9.84); the majority were administrative employees (57.5%), 26.5% were blue-collars, 14.6% were managers, and 1.4% had other occupations. The education level of the sample was rather high with 81.4% of participants having a high-school or university degree. The majority of participants (95.9%) had a full-time contract (40 hours per week) and a seniority in their company of up to ten years (63.4%; over ten years 36.6%).

Participants were recruited in four different companies (operating in the oil and gas industry and the metalworking sector). The study was approved by the Ethical Committee for the Psychological Research of the University of Padova. All participants gave written informed consent and were duly informed that participation in was anonymous and voluntary.

#### Measures

The 18 items of the Spirit at Work Scale (SAWS) [3, 43] were administered to evaluate workplace spirituality. The instrument assesses the experience of spirituality in the workplace through four scales: engaging work (e.g., “I am passionate about my work”), sense of community (e.g., “I feel like I am part of ‘a community’ at work”), spiritual connection (e.g., “My spiritual beliefs play an important role in the everyday decisions that I make at work”), and mystical experience (e.g., “I experience moments at work where everything is blissful”). The items were scored on a 6-point scale ranging from 1 “completely untrue” to 6 “completely true”. In the current study, reliability coefficients were satisfactory for all scales (alphas ranging from .61 to .87; .91 for the total scale score).

Work engagement was assessed using the shortened Italian version of the Utrecht Work Engagement Scale (UWES-9) [99, 100]. The instrument comprises three subscales, with three items each: vigor (e.g., “At my job, I feel strong and vigorous”), dedication (e.g., “My job inspires me”), and absorption (e.g., “I feel happy when I am working intensely”). Answers were recorded on a 6-point scale, from 1 “completely untrue” to 6 “completely true”. In the current study, alpha coefficients were satisfactory for all scales (alphas ranging from .79 to .91; .92 for total scale score).

The 10 items of the Italian version of the Positive Affect Scales (taken from PANAS) [101, 102] were used to evaluate positive affectivity. The instrument assesses the extent to which positive affective states (PA) are generally experienced by individuals. Answers were rated on a 5-point scale ranging from 1 “very slightly or not at all” to 5 “extremely”. In this study alpha coefficient was .89.

The nine items of the Self-efficacy scale [44] were administered to measure self-efficacy. Answers were recorded on a 6-point scale, from 1 “completely untrue” to 6 “completely true”. In the current study, the alpha reliability coefficient was satisfactory (alpha .81).

Resilience was evaluated through the ten items of the Resilience scale [44]. The scale assesses the extent to which individuals remain focused on their goals and inclination to effectively cope with difficulties. Items were scored on a 6-point scale, from 1 “completely untrue” to 6 “completely true”. High scores on this scale indicate a higher degree of resilience. The alpha coefficient in this study was satisfactory (alpha = .85).

#### Analysis strategy

LPA was run on the responses to the four subscales of SAWS. To perform LPA, the means of the four SAWS scales were entered into the analysis and four models were run one after another and compared. Specifically, the one-class model was run first and compared with all models up to four classes. To identify the best fitting model, several statistics were examined: Bayesian Information Criterion (BIC) [103], Sample-Adjusted BIC (SABIC) [104], Akaike’s Information Criterion (AIC) [105], and Entropy. Concerning AIC, BIC, and SABIC, lower values indicate a better fit [106]. In contrast, for entropy, a good fit is suggested by higher values. This latter index defines how well a model classifies individuals into the derived profiles. Entropy ranges from 0 to 1, and values close to 1 indicate a good fit [107]. Additionally, models were compared using the Vuong-Lo-Mendell-Rubin likelihood ratio test (VLMR) [106] and the Lo-Mendell-Rubin likelihood ratio test (adjusted LMR) [108]. These tests are used to compare a model with *C* latent classes against a model with *C*– 1 classes. Significant *p*-values indicate that the model with *C* classes fits the data better than the more parsimonious model (i.e., with one fewer class). On the contrary, non-significant *p*-values suggest retaining the more parsimonious one. Finally, the interpretability of the solution was also considered in the selection of the best model [94].

Student’s *t* statistics were used to test mean score differences across classes, on positive affectivity, resilience, self-efficacy, and work engagement. Eta squared was used as a measure of effect size.

All the analyses were run using the Mplus7.4 and SPSS statistical packages [80, 109].

### Results

Table 2 provides fit indices of the LPA. Results indicate that the model with two classes is the best fitting. Although the AIC, BIC, and SABIC values decreased as the number of classes increased, the VLMR and LMR tests indicated that the two-class model should be preferred to the less parsimonious ones (i.e., models with three and four classes). The entropy value for the two-class model was satisfactory.

**Table 2:** Fit statistics for LPA models.

	1 Class	2 Classes	3 Classes	4 Classes
**AIC**	2547.76	2257.05	2176.46	2155.31
**BIC**	2575.33	2301.86	2238.50	2234.59
**SABIC**	2549.98	2260.65	2181.45	2161.69
**Entropy**		0.83	0.85	0.90
**VLMR**		300.71	90.59	31.14
***p*-Value**		< .001	0.08	0.13
**LMR**		290.06	87.38	30.04
***p*-Value**		< .001	0.08	0.14

Note: AIC: Adjusted Bayesian information criterion; BIC: Bayesian information criterion; SABIC: Sample-Adjusted BIC; VLMR: Vuong-Lo-Mendell-Rubin; LMR: Adjusted Lo-Mendell-Rubin.

**Fig 2:**
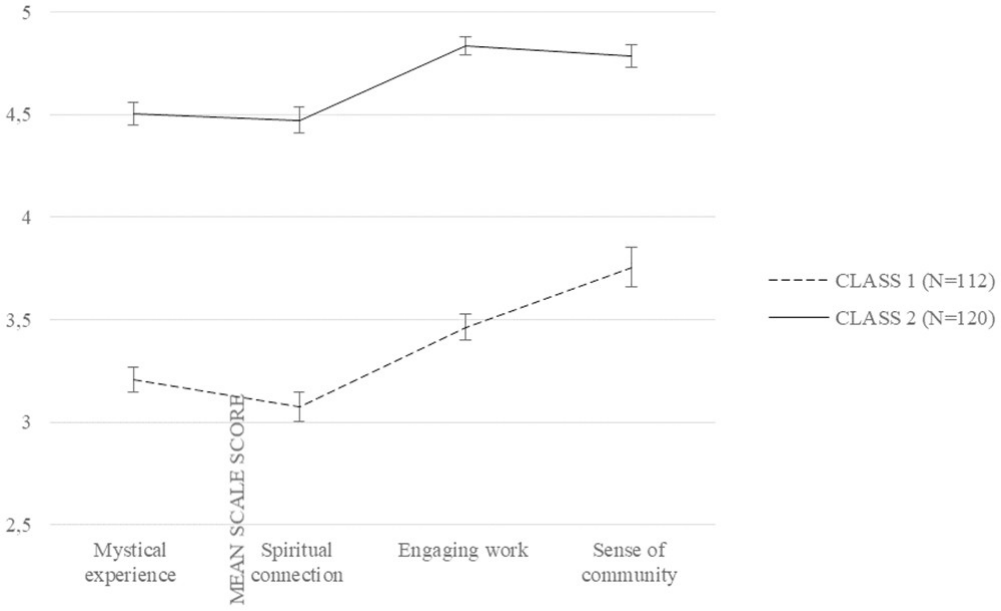
Means SAWS scales in the two classes from LPA. Note. The graph represents mean scores on SAWS scales on the two classes identified through LPA.

Fig 2 reports mean scores on the four SAWS scales for the two identified classes. The pattern of scores is similar in both groups, with lower scores for mystical experience and spiritual connection, and higher for engaging work and sense of community. However, the two profiles significantly differed in the mean scores in each scale (*t* (230) = -15.68, *η*^*2*^ = .52; *t* (230) = -14.49, *η*^*2*^ = .48; *t* (230) = -17.77, *η*^*2*^ = .58; *t* (230) = -9.40, *η*^*2*^ = .28; *p*s ≤ .001, for mystical experience, spiritual connection, engaging work, and sense of community, respectively). Specifically, when compared with the second one, the first class is characterized by lower scores on all scales. In other words, the two profiles seem to describe individuals characterized by a low (first class) or a high (second class) workplace spirituality profile.

To further explore the meaning of the profiles emerged, Student’s *t* was used. T-tests were run to explore the mean score differences across the two classes on positive affectivity, resilience, self-efficacy, and work engagement. Table 3 reports our findings. Participants of the two classes differed on all the variables considered. In particular, individuals falling into the high workplace spirituality profile (i.e., second class) experience greater positive affectivity, resilience, self-efficacy, vigor, dedication, absorption, and work engagement-total score. Effect sizes were all medium to large but the stronger effects were found for the work engagement dimensions and positive affectivity.

**Table 3:** Descriptive statistics and t-test.

		Mean score	SD	t-test
**Positive affectivity**	CLASS 1	3.08	0.58	(*t* (230) = -7.63, *p* ≤ .001; *η*^*2*^ = .20)
	CLASS 2	3.71	0.66	
**Resilience**	CLASS 1	4.50	0.62	(*t* (230) = -6.06, *p* ≤ .001; *η*^*2*^ = .14)
	CLASS 2	4.93	0.48	
**Self-efficacy**	CLASS 1	4.82	0.66	(*t* (230) = -3.49, *p* ≤ .001; *η*^*2*^ = .05)
	CLASS 2	5.13	0.69	
**Vigor**	CLASS 1	3.60	0.86	(*t* (230) = -10.50, *p* ≤ .001; *η*^*2*^ = .32)
	CLASS 2	4.63	0.62	
**Dedication**	CLASS 1	3.64	0.93	(*t* (230) = -12.04, *p* ≤ .001; *η*^*2*^ = .39)
	CLASS 2	4.93	0.69	
**Absorption**	CLASS 1	3.92	0.83	(*t* (230) = -9.90, *p* ≤ .001; *η*^*2*^ = .30)
	CLASS 2	4.92	0.71	
**Work engagement-total score**	CLASS 1	3.72	0.71	(*t* (230) = -13.11, *p* ≤ .001; *η*^*2*^ = .43)
	CLASS 2	4.83	0.57	

Note: *η*^*2*^ = eta squared.

### Discussion

This study aimed to explore the relations of workplace spirituality with a series of variables relevant in organizational contexts. In addition, LPA was run to identify groups of individuals characterized by a similar workplace spirituality profile. Results showed that two main classes may be recognized, namely the low workplace spirituality profile and the high workplace spirituality profile. Overall, the two groups showed a similar pattern of scores on the four scales of the SAWS and are characterized by higher feelings of engaging work and sense of community, and lower scores on the two dimensions of mystical experience and spiritual connection. However, the two classes differed remarkably on intensity of workplace spiritual experience. Moreover, the two profiles are differently associated with a series of variables, which have a relevant role in organizational contexts. Specifically, individuals falling into the high workplace spirituality profile showed greater positive affectivity, resilience, self-efficacy, and work engagement, if compared with the individuals of the low workplace spirituality profile. These results suggest that spirituality may have a crucial role in the organizational field because it may influence several subjective experiences, which, in turn, could affect individual and organizational well-being.

## General discussion

Since ancient times, spirituality has been a fascinating theme and an intrinsic need for all human beings [110]. However, only recently it has been scientifically operationalized so as to be effectively investigated across different areas of human existence. For instance, in the last few years the construct has gained increasing attention in the organizational research field, where it has been named workplace spirituality or spirit at work − terms used as synonyms in this paper [3, 8, 29, 111]. Overall, the literature on the topic converges in attributing a positive effect of the construct on both organizational goals and employee well-being [22, 23, 26].

The two studies of this paper are in line with the findings of the literature. In particular, the first study confirmed that individual characteristics are fundamental to prevent burnout [112]. Specifically, it has been found that workplace spirituality has a positive effect in reducing employee burnout; moreover, it also mediates the positive effect of supervisor integrity and responsible behaviors in reducing this threat. In other words, these results indicate that supervisor integrity and positive behaviors have a beneficial effect on employee well-being as long as they favor feelings of spirit at work. These findings, thus, highlight the value of developing burnout prevention programs which take into account the employees’ spiritual needs. The results also indicate the usefulness of developing specific training programs for supervisors aimed to increase their abilities to show integrity and consideration for employees’ needs.

These results have been extended in Study 2. The study, in particular, showed that workplace spirituality is associated not only with employee well-being, but also with other subjective feelings, such as positive affectivity, resilience, self-efficacy, and work engagement. The study specifically highlighted the existence of two main workplace spirituality profiles, which are characterized by an analogous pattern of mean scores in the four main dimensions of the construct but by a different level of intensity of spiritual experience. The two profiles, in addition, differ in their associations with a series of variables that are relevant in the organizational field. Specifically, the findings of this study showed that the participants who fall into the high workplace spirituality profile report higher positive affectivity, resilience, self-efficacy, and work engagement. The results of this study, therefore, indicate that spirit at work has a relevant role in several areas of working life, which affect both organizational well-being and global well-being of employees [113].

The findings of the present work suggested that efforts should be devoted to developing intervention programs for improving supervisor behavioral competencies [114, 115], because supervisor skills and his/her ability in shaping working environment have considerable effects on employee and organizational well-being [116]. In particular, interventions should involve the whole organization, from senior management to line managers, and focus on the recognition of employees’ spiritual needs. Creating an organizational culture responsive to workplace spirituality catalyzes the acceptance, adhesion, and effectiveness of this kind of intervention. Internalization of workplace spirituality practices into human resource management is allowed only by this spirit-at-work awareness. The importance of being spiritual at work is already recognized in healthcare, but it could be enhanced in other work contexts as well. In fact, finding these results in the work sectors considered in the present work is rather innovative. Moreover, the workplace spirituality profiles identified combine perfectly with the aims of positive organizations—workplaces genuinely devoted to organizational development and well-being, corporate social responsibility, enhancement of working conditions, and prevention of work-related stress [31, 117].

Globally, the results of this paper highlighted the importance of workplace spirituality in organizational contexts and its effectiveness in conditioning the work experience. The results are promising and help to thoroughly understand the construct, its correlates and features. However, some limitations can be identified, such as the cross-sectional nature of the research. Future studies should use longitudinal designs and try to replicate our results in cross-cultural contexts. They may also include objective measurements or observer ratings to assess correlates of spirit at work [118]. Finally, future research could investigate the association between workplace spirituality and passion for work—e.g., harmonious and obsessive passion [119]–or negative forms of heavy work investment—e.g., workaholism [120].

## Supporting information

S1 Dataset (TXT)

S2 Dataset (TXT)

S1 File (TXT)

S2 File (TXT)
